# Editorial: Enhancing patient outcomes after cataract, corneal and refractive surgery

**DOI:** 10.3389/fmed.2025.1697162

**Published:** 2025-10-03

**Authors:** Mayank A. Nanavaty, Ramin Khoramnia

**Affiliations:** ^1^Sussex Eye Hospital, University Hospitals Sussex NHS Foundation Trust, Brighton, United Kingdom; ^2^University Hospital Carl Gustav Carus, TU Dresden, Dresden, Germany

**Keywords:** cataract surgery, corneal surgery, refractive surgery, intraocular lenses, extended depth of focus, patient outcomes, surgical techniques, visual outcomes

The field of ophthalmic surgery continues to evolve at an unprecedented pace, driven by technological advances, refined surgical techniques, and an increasingly sophisticated understanding of patient-centered care. The Research Topic titled “*Enhancing patient outcomes after cataract, corneal and refractive surgery*” published in Frontiers in Medicine presents thirteen carefully curated studies that collectively illuminate the current state of these surgical disciplines and point toward future directions for improving patient outcomes. These contributions span the entire spectrum of contemporary ophthalmic surgical practice, from advanced intraocular lens technologies to novel surgical techniques and comprehensive outcome assessments.

## Contemporary advances in intraocular lens technology

### Extended depth of focus and enhanced monofocal IOLs

The evolution of intraocular lens technology represents one of the most significant advances in modern cataract surgery, with particular emphasis on providing patients with extended ranges of functional vision while minimizing photic phenomena. The Research Topic includes pivotal research on non-diffractive enhanced depth-of-focus (EDOF) IOLs, which demonstrate remarkable potential in challenging patient populations. The work by Elvira and colleagues examining visual outcomes with non-diffractive EDOF IOLs in patients with age-related macular degeneration represents a paradigm shift in surgical decision-making for this complex population. Recent evidence suggests that patients with AMD who undergo cataract surgery with EDOF IOL implantation can achieve functional spectacle-free vision ranges while maintaining contrast sensitivity within acceptable parameters. These findings are particularly significant given that traditional teaching has advocated for monofocal IOLs in AMD patients due to concerns about visual quality degradation (Elvira et al.) (1).

Complementing this research, the comprehensive review by Levy and colleagues on mini-monovision outcomes with monofocal, enhanced monofocal, and EDOF lenses provides crucial clinical guidance for optimizing presbyopia correction strategies. The systematic analysis demonstrates that mini-monovision with EDOF IOLs achieves spectacle independence rates of 63.4% compared to 51% and 55% for monofocal and enhanced monofocal lenses, respectively (Levy et al.). This approach offers superior intermediate and near visual acuity while maintaining excellent distance vision, with a mean logMAR binocular uncorrected intermediate visual acuity of 0.08 ± 0.07 (Levy et al.).

### Post-refractive surgery considerations

The increasing prevalence of patients with previous refractive surgery presenting for cataract extraction has necessitated specialized approaches to IOL selection and power calculation. The investigation by Fan and colleagues on wavefront-shaping intraocular lenses in post-LASIK patients demonstrates that modern EDOF IOLs can provide excellent visual outcomes in this challenging population. Post-LASIK eyes achieved superior uncorrected near visual acuity compared to virgin eyes, with defocus curves maintaining visual acuity close to 0.3 logMAR even at −3.0 diopters (Fan et al.).

This superior performance in post-LASIK eyes may be attributed to the interaction between residual corneal higher-order aberrations and the wavefront-shaping properties of modern EDOF lenses, creating a synergistic effect that enhances depth of focus. Such findings challenge traditional assumptions about visual quality in post-refractive surgery patients, suggesting that appropriately selected presbyopia-correcting IOLs may offer significant advantages over conventional monofocal lenses (Fan et al.) (2, 3).

## Precision in IOL power calculation

### Intraoperative aberrometry vs. preoperative biometry

The quest for refractive predictability has led to significant advances in IOL power calculation methodologies, particularly for challenging cases, such as those with short and long eyes. The comparative study by Tañá-Rivero and colleagues examining intraoperative aberrometry vs. preoperative biometry represents a significant contribution to understanding optimal approaches for IOL power selection in extreme axial lengths (Tañá-Rivero et al.).

Contemporary research demonstrates that intraoperative aberrometry exhibits superior performance in eyes with long axial lengths (>25.0 mm) compared to traditional biometry-based formulas, with significantly lower mean absolute errors and reduced instances of hyperopic surprises. In short eyes (<22.1 mm), intraoperative aberrometry performs comparably to the most advanced biometry-based formulas, including Barrett Universal II and Hill-RBF, suggesting its utility as a valuable adjunctive tool rather than a replacement for sophisticated preoperative calculations (Tañá-Rivero et al.) (4, 5). The clinical implications are substantial, as accurately calculating IOL power in extreme axial lengths remains one of the most significant challenges in modern cataract surgery.

## Surgical technique innovations

### Advanced techniques for complex cases

The management of complex cataracts continues to challenge even experienced surgeons, necessitating innovative approaches to minimize complications and optimize outcomes. The clinical trial by Huang and colleagues, investigating the artificial lens cushion plate technique for hard-core cataracts, demonstrates significant advances in protecting the corneal endothelium during challenging cases (Huang et al.). This technique demonstrates remarkable efficacy in preserving corneal endothelial cells, with significantly lower endothelial cell loss rates compared to conventional phacoemulsification (*p* < 0.05). The reduced ultrasonic energy requirements and decreased total energy consumption associated with this approach represent important advances in managing the most challenging nuclear densities while maintaining surgical safety (Huang et al.) (6).

## Astigmatism management and tolerance

### Understanding premium IOL performance with residual astigmatism

The comprehensive analysis by Mu and colleagues examining astigmatism tolerance in patients with trifocal and EDOF IOLs provides crucial clinical insights for optimizing patient selection and managing expectations (Mu et al.). The study demonstrates that both EDOF and trifocal lenses show reduced tolerance for oblique astigmatism compared to with-the-rule or against-the-rule astigmatism, with EDOF lenses generally demonstrating superior objective visual quality regardless of astigmatism magnitude or axis (Elvira et al.; Mu et al.). These findings have significant implications for surgical planning, suggesting that astigmatism correction should be prioritized when considering presbyopia-correcting IOLs, particularly when residual astigmatism exceeds −1.00 diopter. The differential tolerance patterns between lens types provide valuable guidance for IOL selection in patients with varying degrees of corneal astigmatism (Mu et al.).

## Surgical training and competency

### Resident education and patient safety

The evaluation of surgical training outcomes represents a critical component of maintaining high standards in ophthalmic surgery. The comparative study by Wu and colleagues examining phacoemulsification outcomes between resident and attending physicians provides valuable insights into surgical education and patient safety (Wu et al.).

Contemporary research demonstrates that resident-performed phacoemulsification can achieve excellent visual outcomes comparable to attending-performed surgery, with over 95% of patients achieving 20/40 or better vision. However, the learning curve analysis reveals that surgical competency continues to improve well beyond the first 80 cases, with significant reductions in complication rates and improved surgical efficiency occurring throughout residency training (Wu et al.) (7, 8). The implications for residency training programs are substantial, with recent increases in minimum case requirements from 45 to 86 procedures appearing well-justified based on learning curve data. These findings support the importance of structured surgical curricula, adequate supervision, and sufficient case volume in developing competent cataract surgeons (8).

## Complications and management strategies

### Corneal epithelial healing complications

The comprehensive case series by Yan and colleagues, addressing delayed corneal epithelial healing after refractive surgery, highlights an important but underrecognized complication that can significantly impact patient outcomes (Yan et al.). The management strategies presented, including the use of amniotic membrane transplantation in severe cases, provide valuable clinical guidance for managing this challenging complication (Yan et al.).

Recent systematic reviews indicate that epithelial healing complications occur in 0.02–17.1% of refractive surgery cases, with higher rates associated with photorefractive keratectomy compared to LASIK. Risk factors include prolonged contact lens wear, previous ocular surface disease, and certain surgical techniques, emphasizing the importance of careful preoperative assessment and patient counseling (9, 10).

## Emerging technologies and assessment methods

### Advanced imaging and measurement techniques

The comparative analysis by Ning and Zhang examining different topographic measurement systems for pupil offset assessment in myopic populations demonstrates the continuing evolution of preoperative assessment technologies (Ning et al.). The integration of Scheimpflug tomography, Placido disc, and combined systems provides increasingly sophisticated approaches to characterizing corneal and anterior segment anatomy (Ning et al.).

These advances in imaging technology are particularly relevant for refractive surgery planning and IOL selection, where precise characterization of corneal irregularities and optical aberrations is crucial for optimizing outcomes. The ability to accurately measure pupil dynamics and centration parameters has significant implications for the performance of presbyopia-correcting IOLs and patient satisfaction.

## Psychosocial impact and quality of life

### Mental health considerations in cataract surgery

The cross-sectional study by Wang and colleagues examining the relationship between untreated cataracts and depression symptoms provides important insights into the broader impact of visual impairment on patient well-being (Wang et al.). The demonstration that age-related cataracts without surgical intervention are associated with exacerbated depression symptoms underscores the importance of timely surgical intervention and comprehensive patient care (Wang et al.). Contemporary research consistently demonstrates that successful cataract surgery not only improves visual function but also has significant positive impacts on quality of life, mental health, and overall patient wellbeing. These findings support the concept that cataract surgery should be considered not merely as a vision-restoring procedure but as a comprehensive intervention with broad health and social benefits (11, 12).

## Future directions and research trends

### Bibliometric analysis and research evolution

The bibliometric analysis by Zhang and colleagues examining trends in implantable collamer lens surgery research provides valuable insights into the evolution and future directions of refractive surgery research (Zhang et al.). The analysis reveals an increasing interest in ICL surgery as a safe and effective alternative to corneal refractive procedures, particularly for correcting high myopia (Zhang et al.) (13). Current trends indicate growing emphasis on patient-reported outcomes, long-term safety profiles, and optimization of surgical techniques for different patient populations. The continuous refinement of ICL designs, including the development of central hole technology, eliminates the need for peripheral iridotomy, representing significant advances in patient safety and surgical convenience (Zhang et al.) (13, 14).

## Clinical implications and recommendations

Based on the comprehensive evidence presented in this Research Topic, several key clinical recommendations emerge:

### IOL selection strategy

Enhanced monofocal and EDOF IOLs with mini-monovision approaches offer excellent alternatives to traditional monofocal lenses, providing improved spectacle independence while maintaining acceptable visual quality. In post-refractive surgery patients, wavefront-shaping IOLs may provide superior outcomes compared to conventional lenses (Levy et al.; Fan et al.) (15).

### Astigmatism management

Residual astigmatism exceeding −1.00 diopter should be addressed surgically when implanting presbyopia-correcting IOLs, with particular attention to oblique astigmatism, which is less well-tolerated than with-the-rule or against-the-rule astigmatism (Mu et al.) (16).

### Surgical training

Residency programs should ensure adequate case volume and structured training curricula to optimize learning curves and patient safety. The learning curve for phacoemulsification extends well beyond initial case requirements, emphasizing the importance of ongoing skill development (7, 8).

### Complication management

Early recognition and aggressive management of epithelial healing complications following refractive surgery are crucial for preventing long-term sequelae. Amniotic membrane transplantation represents an effective treatment option for severe cases resistant to conventional therapy (Yan et al.).

## Conclusion

The Research Topic “*Enhancing patient outcomes after cataract, corneal and refractive surgery*” provides compelling evidence of the rapid evolution occurring in ophthalmic surgery. From advanced IOL technologies that provide excellent outcomes in challenging patient populations to innovative surgical techniques that minimize complications, these contributions represent significant advances in our ability to optimize patient care.

The integration of precise preoperative assessment, advanced surgical techniques, and comprehensive outcome evaluation creates a framework for evidence-based practice that prioritizes both visual function and patient satisfaction. As we continue to refine these approaches, the ultimate goal remains unchanged: providing each patient with the safest, most effective surgical intervention that optimizes their individual visual needs and quality of life.

The future of cataract, corneal, and refractive surgery lies in the continued evolution of personalized medicine approaches, where advanced technologies, refined surgical techniques, and comprehensive patient assessment combine to deliver truly customized surgical solutions. The research presented in this Research Topic provides an excellent foundation for this ongoing evolution, offering both immediate clinical applications and direction for future investigation.
